# A novel allele of *TaGW2*-*A1* is located in a finely mapped QTL that increases grain weight but decreases grain number in wheat (*Triticum aestivum* L.)

**DOI:** 10.1007/s00122-017-3017-y

**Published:** 2017-11-17

**Authors:** Huijie Zhai, Zhiyu Feng, Xiaofen Du, Yane Song, Xinye Liu, Zhongqi Qi, Long Song, Jiang Li, Linghong Li, Huiru Peng, Zhaorong Hu, Yingyin Yao, Mingming Xin, Shihe Xiao, Qixin Sun, Zhongfu Ni

**Affiliations:** 1 State Key Laboratory for Agrobiotechnology, Key Laboratory of Crop Heterosis and Utilization, Beijing Key Laboratory of Crop Genetic Improvement, China Agricultural University, Beijing, 100193 China; 2 National Plant Gene Research Centre, Beijing, 100193 China; 3 Millet Research Institute, Shanxi Academy of Agricultural Sciences, Changzhi, 046011 Shanxi China; 4 Institute of Crop Science, Chinese Academy of Agricultural Sciences, Beijing, 100081 China

## Abstract

**Key message:**

A novel *TaGW2*-*A1* allele was identified from a stable, robust QTL region, which is pleiotropic for thousand grain weight, grain number per spike, and grain morphometric parameters in wheat.

**Abstract:**

Thousand grain weight (TGW) and grain number per spike (GNS) are two crucial determinants of wheat spike yield, and genetic dissection of their relationships can help to fine-tune these two components and maximize grain yield. By evaluating 191 recombinant inbred lines in 11 field trials, we identified five genomic regions on chromosomes 1B, 3A, 3B, 5B, or 7A that solely influenced either TGW or GNS, and a further region on chromosome 6A that concurrently affected TGW and GNS. The QTL of interest on chromosome 6A, which was flanked by *wsnp_BE490604A_Ta_2_1* and *wsnp_RFL_Contig1340_448996* and designated as *QTgw/Gns.cau*-*6A*, was finely mapped to a genetic interval shorter than 0.538 cM using near isogenic lines (NILs). The elite NILs of *QTgw/Gns.cau*-*6A* increased TGW by 8.33%, but decreased GNS by 3.05% in six field trials. *Grain Weight 2* (*TaGW2*-*A1*), a well-characterized gene that negatively regulates TGW and grain width in wheat, was located within the finely mapped interval of *QTgw/Gns.cau*-*6A*. A novel and rare *TaGW2*-*A1* allele with a 114-bp deletion in the 5′ flanking region was identified in the parent with higher TGW, and it reduced *TaGW2*-*A1* promoter activity and expression. In conclusion, these results expand our knowledge of the genetic and molecular basis of TGW-GNS trade-offs in wheat. The QTLs and the novel *TaGW2*-*A1* allele are likely useful for the development of cultivars with higher TGW and/or higher GNS.

**Electronic supplementary material:**

The online version of this article (10.1007/s00122-017-3017-y) contains supplementary material, which is available to authorized users.

## Introduction

Wheat provides approximately 20% of the calories consumed by humankind (Simmonds et al. 2016). Considering the continued global population growth and the low rates of genetic gain in wheat yield (Zheng et al. 2011; Ray et al. 2013), there is an urgent need to identify, characterize, and incorporate genomic tools that can accelerate wheat yield improvement (Simmonds et al. 2016). Wheat yield is controlled by polygenes and affected by environmental factors, and it mainly relies on three components: thousand grain weight (TGW), grain number per spike (GNS), and spike number per hectare (Simmonds et al. 2014). There are negative correlations among these components (Griffiths et al. 2015), but the genetic basis underlying individual yield traits and their interactions are still largely unknown in wheat.

In rice, it has been shown that grain weight is affected by the genes that functioning in several pathways, i.e., proteasomal degradation (*GW2* and *GW5*/*qSW5*), phytohormone signaling (*GS6*, *TGW6,* and *OsCKX2*), and G protein-mediated signal transduction (*GS3* and *RGB1*); these genes regulate cell division and/or cell expansion in specific grain tissues (reviewed by Zuo and Li 2014). Using a homology-based approach, several wheat genes have been isolated and suggested to be associated with TGW. These include *TaCwi* (Jiang et al. 2015b; Ma et al. 2012), *TaTGW6* (Hanif et al. 2016; Hu et al. 2016), *TaGW2*-*A1* (Simmonds et al. 2016; Su et al. 2011; Yang et al. 2012), *TaGS1a* (Guo et al. 2013), *TaGS5*-*3A* (Ma et al. 2016), *TaGASR7*-*A1* (Dong et al. 2014; Ling et al. 2013) and *TaCYP78A3* (Ma et al. 2015). Research on these genes has enhanced our understanding on grain weight determination in wheat and has also provided functional markers useful for selecting higher TGW through marker-assisted breeding.

Several major QTLs for grain number have also been isolated and characterized in rice. Some of these QTLs control inflorescence meristem identity (e.g., *LAX1*, *SPA,* and *FZP*), while others influence the rate (e.g., *APO1*, *Gn1a*/*OsCKX2*, *LOG*, *SP1,* and *DEP1*) or duration (e.g., *RCN1*, *RCN2*, *Ghd7,* and *Ghd8*) of cell division in the inflorescence meristem (reviewed by Xing and Zhang 2010). Although the functions of orthologous genes are generally conserved in rice and wheat, there are several examples of functional divergence between rice grain number genes and their wheat homologs. For instance, *OsCKX2*, encoding a cytokinin oxidase, is a negative regulator of the number of grains per panicle in rice (Ashikari et al. 2005). On the other hand, the two wheat homologs of *OsCKX2*, i.e., *TaCKX2.1* and *TaCKX2.2*, have been suggested to positively control GNS (Zhang et al. 2011). Furthermore, while *MOC1* and *OsTEF1* are two crucial regulators of rice tillering (Li et al. 2003; Paul et al. 2012), their wheat homologs (*TaMOC1*-*7A* and *TaTEF*-*7A*) have both been found to be stably associated with spikelet number per spike rather than with tiller number (Zhang et al. 2015; Zheng et al. 2014). Differences such as these may be associated with the contrasting architecture of the inflorescences between wheat (spikes) and rice (panicles).

Here, we report mapping of several stable QTLs for TGW and/or GNS using 191 recombinant inbred lines (RILs) derived from a cross between two wheat lines that differ in both TGW and GNS. Of these QTLs, the one on chromosome 6A displayed a strong TGW-GNS trade-off. This QTL was further validated and precisely mapped using near isogenic lines (NILs). A novel allele of *TaGW2*-*A1* was isolated from the finely mapped interval, which is likely a candidate.

## Materials and methods

### Plant materials

A population of 207 RILs was developed from the cross between two Chinese hexaploid winter wheat cultivars, i.e., Yumai 8679 (Y8679) and Jing 411 (J411), and advanced to the F_9_ generation by single seed descent. Of the 207 RILs, 191 were genotyped and evaluated in 11 field trials. An F_9_ plant of the RIL line (RIL186) with residual heterozygosity at the marker locus *Xbarc118* was selfed and provided an F_9:10_ family with 163 progeny. Afterwards, selfing of these plants was carried out to obtain a population of 163 F_10:11_ families. Four sets of NIL pairs (NIL1, NIL2, NIL3, and NIL4) were obtained by selfing selected F_11_ progeny with overlapping heterozygous fragments. Each NIL set comprised eight to 25 Y8679-type homozygotes and 12–30 J411-type homozygotes.

In total, 1113 wheat accessions with varying ploidy were used to test the allele frequency of a novel *TaGW2*-*A1* allele. These included 848 hexaploid wheat accessions (96 Chinese landraces, 702 Chinese modern cultivars, and 50 French varieties; Supplementary Table S1), 238 tetraploid accessions (181 *Triticum dicoccoides*, 33 *Triticum durum,* and 24 *Triticum dicoccum*; Supplementary Table S2), and 27 diploid accessions (6 *Triticum urartu*, 6 *Triticum boeoticum,* and 15 *Triticum monococcum*; Supplementary Table S3).

### Field experiments and phenotyping

The RIL population was grown in three replicates following randomized complete-block design at the following five experimental sites: Anhui (33°48′N, 116°35′E), Beijing (40°08′N, 116°10′E), Hebei (37°56′N, 114°42′E), Shaanxi (34°17′N, 108°04′E), and Shanxi (36°08′N, 111°34′E) (Supplementary Fig. S1; Supplementary Table S4). The field trials were carried out during two crop seasons (2011–12 and 2012–13) at Anhui, four crop seasons (2010–11, 2011–12, 2012–13, and 2014–15) at Beijing, three crop seasons (2011–12, 2012–13 and 2013–14) at Shaanxi, and one crop season (2013–14 or 2014–15) at the other two sites. For the field trials, the seeds were sown in double-row plots (2 m long) at a sowing rate of 30 seeds per row. At maturity, 20 representative spikes (from 20 different plants) were sampled from each plot and threshed together. For the grain samples obtained from the first two crop seasons (2010–11 and 2011–12), GNS, TGW, and grain weight per spike (GWS) were manually assessed. For the samples obtained from the other three seasons (2012–13, 2013–14, and 2014–15), seven traits [i.e., GNS, TGW, GWS, grain surface area (GA), grain circumference (GC), grain length (GL), and grain width (GW)] were recorded using a scaled camera-assisted phenotyping system (Wanshen Detection Technology Co., Ltd., Hangzhou, China). For each RIL line, the mean value of each trait was calculated across three replicates for each individual environment. In addition, best linear unbiased prediction (BLUP) values were predicted for each trait using the PROC MIXED procedure in SAS v9.1.3 (SAS Institute Inc., North Carolina, USA). Pearson’s correlation coefficient was calculated using SPSS v20.0 (SPSS, Chicago, USA) to assess the correlation among TGW, GNS, GWS, and grain morphometric parameters. The Shapiro–Wilk test was performed using R v3.2.2 to identify departures from normal distribution. Broad sense heritability ($h_{\text{B}}^{2}$) based on a family mean basis was calculated using the PROC GLM procedure in SAS according to the following formula: $h_{\text{B}}^{2} = \sigma_{\text{g}}^{2} /(\sigma_{\text{g}}^{2} + \sigma_{\text{ge}}^{2} /n + \sigma^{2} /nr)$, where $\sigma_{\text{g}}^{2}$ is the genotypic effect, $\sigma_{\text{ge}}^{2}$ is the genotype by environmental effect, *σ* ^2^ is the residual error, *n* is the number of environments and *r* is the number of replicates (Liu et al. 2014).

The NIL pairs were evaluated at three sites (Beijing, Hebei, and Shanxi) in crop seasons 2014–15 (NIL1) and 2015–16 (NIL1, NIL2, NIL3, and NIL4). The 163 F_10:11_ families were evaluated at Beijing during crop season 2014–15. These NIL pairs and families were grown in single-row plots (2 m long and 30 seeds per row) in randomized complete-block designs with three replicates. At maturity, 20 representative spikes were sampled, and the GWS, GNS, TGW, and grain morphometric parameters (GA, GC, GL, and GW) were assessed. For the members of NIL1 pair, tiller number (TN) and grain weight per plant (GWP) were determined for each of ten representative plants. The significance of phenotypic variations between NIL1-Y8679 and NIL1-J411 was calculated using Student’s *t* test.

### Linkage map and QTL analysis

The Y8679/J411 linkage map used here is the one that was described by Zhai et al. (2016). It includes both single-nucleotide polymorphism (SNP) markers and simple sequence repeat (SSR) markers. QTL analysis was conducted using both the within-environment means and the across-environment BLUPs for each trait. The methods used for QTL analysis were the same as those used by Zhai et al. (2016) for other traits in the same population. Briefly, this involved the use of WinQTLCart version 2.5 (Wang et al. 2012) for composite interval mapping (CIM) using model 6 with forward and backward regression, five markers as cofactors, and a 10-cM scanning window. Empirical threshold LOD scores estimated with 1000 permutations at *P* ≤ 0.05 were used to declare a significant QTL (Churchill and Doerge 1994). Detected QTLs with overlapping confidence intervals (± 2 LOD away from the peaks of likelihood ratios) were considered equivalent and named as suggested by McIntosh et al. (2011).

For a QTL of interest on chromosome 6A, the 163 F_10:11_ families derived from RIL186 were assigned to three genotypic classes (two homozygous families and one segregating families) based on their marker genotypes. This classification was used to estimate the additive effect (*a*), dominance effect (*d*), and dominance degree (*d*/*a*) (Falconer and Mackay 1996). Multiple comparisons among the mean values were estimated by the LSD method. The inheritance mode of an individual QTL can be classified into four categories, i.e., additive (*d/a* ≤ 0.20), partial dominance (0.20 < *d/a* < 0.80), dominance (0.80 ≤ *d/a* < 1.20), and overdominance (*d/a* ≥ 1.20), as described in the previous studies (Jiang et al. 2015a; Li et al. 2017).

The SNPs mapped in the QTL regions of interest were positioned onto the newly released reference genome sequence of Chinese Spring by blasting their flanking sequences against the IWGSC RefSeq v1.0 (https://urgi.versailles.inra.fr/blast_iwgsc/blast.php).

### SSR marker development

Polymorphic SNP markers flanking the QTL region of interest were used to perform a BLAST search against genomic sequences of *Brachypodium*, rice, and *Aegilops tauschii*, to identify orthologous genomic regions (Supplementary Table S5) using the methods described by Zhai et al. (2016). The genes within the corresponding regions were used to search the hexaploid wheat cv. Chinese Spring IWGSC survey sequences (http://www.wheatgenome.org) to find homologous contig sequences for marker development (Lu et al. 2016). These contig sequences were further used to search for SSR motifs (with at least 15 dinucleotide or trinucleotide repeats) and design PCR primers using BatchPrimer3 (http://probes.pw.usda.gov/batchprimer3). For polymorphism detection, PCR products were separated on 8% non-denaturing PAGE gels and visualized with silver staining. The primer pairs of 14 co-dominant SSR markers are listed in Supplementary Table S6.

### RNA extraction, cDNA synthesis, and qRT-PCR

For cDNA cloning analysis, the seedling leaves of Y8679 and J411 were collected for total RNA extraction at 7 days after germination (dag). For expression analysis, the immature grains of NIL1-Y8679 and NIL1-J411 were collected for total RNA extraction at 11 days after pollination (dap) using the TransZol Plant Kit (TransGen Biotech). First-strand cDNA was synthesized using M-MLV Reverse Transcriptase (Promega, WI, USA) according to the manufacturer’s instructions.

qRT-PCR was performed to quantify the *TaGW2*-*A1* transcripts in three independent biological repeats using the homoeolog-specific primer pair TaGW2A-Q (Hong et al. 2014) (Supplementary Table S6). qRT-PCR cycling was conducted using SYBR Green PCR master mix (TaKaRa, Japan) on a BioRad CFX96 system (CA, USA) using the following thermal profile: 95 °C for 7 min, 40 cycles at 95 °C for 10 s, 56 °C for 20 s, and 72 °C for 20 s. For each biological sample, three technical replicates were performed for *TaGW2*-*A1* and *Actin* (Supplementary Table S6). After normalizing to the endogenous control, the transcript levels were determined using the 2$^{{ - \Delta {\text{C}}_{t} }}$ method.

### Isolation of full-length *TaGW2*-*A1* cDNA

The complete coding sequences (CDS) of *TaGW2* genes were obtained from the cDNA of Y8679 and J411 using the primer pair TaGW2-1 (Su et al. 2011) (Supplementary Table S6). PCR products were gel-purified and cloned into the pGEM-T Easy Vector (Promega). The identities of three homoeologous coding sequences were determined by blasting against the wheat database (http://plants.ensembl.org/common/Tools/Blast?db=core; Bolser et al. 2015) (Supplementary Fig. S2). The upstream and downstream regions of *TaGW2*-*A1* in J411 were obtained using a rapid amplification of the cDNA ends (RACE) with the GeneRacer Kit (Invitrogen). The gene specific primers for 5′-RACE and 3′-RACE are listed in Supplementary Table S6.

### Promoter isolation and activity analysis of *TaGW2*-*A1*

Three A-genome-specific primer pairs, including Hap-6A-P1 (Su et al. 2011), TaGW2-A1_InDel, and TaGW2-A1_ProF3 (Supplementary Table S6), were used to amplify a region of approximately 2.0-kb upstream from the initiation codon (ATG) in the *TaGW2*-*A1* gene by PCR. The promoters of 1927 and 2041 bp length were obtained from Y8679 and J411, respectively, with a 114-bp deletion existing in the promoter obtained from Y8679.

To assess the effects of this 114-bp deletion on the promoter activity, promoter fragments from Y8679 (1121 bp) and J411 (1235 bp) were amplified (Supplementary Fig. S3) and fused with the β-glucuronidase (GUS) reporter gene sequence in the pBGWFS7.0 vector using the Gateway^®^ system following the manufacturer’s instructions (Invitrogen, CA, USA). The plasmids were transferred into the *Agrobacterium tumefaciens* strain GV3101. The leaves of 7-week-old tobacco (*Nicotiana benthamiana*) plants were infiltrated with the *Agrobacterium* clones. After 3 days, the total protein was extracted from 1.0-cm leaf discs (without the central veins), and the specific GUS activity of these extracts was determined using a colorimetric assay according to Leborgne-Castel et al. (1999). Briefly, the protein extracts were incubated with *p*-nitrophenyl β-d-glucuronide (PNPG) substrate at 37 °C and absorbance of the *p*-nitrophenol (PNP) product was measured at 410 nm (Pawar et al. 2017). Leaves infiltrated with the empty vector were used as controls. Total protein content in the extracts was quantified using the Bradford protein assay.

## Results

### Phenotypic performance of the RIL population

The population means and ranges of the seven investigated traits are listed in Table 1. Compared with J411, Y8679 had higher TGW and GWS, larger grain size (GA, GC, GL, and GW), and lower GNS (Supplementary Fig. S4). TGW displayed an obvious shift towards higher values in the Y8679/J411 RIL population, whereas the other six traits exhibited normal distributions (Supplementary Fig. S5). All traits had broad sense heritabilities over 0.80. Pearson’s correlation coefficients among the seven traits were calculated based on the BLUP values across seven shared environments (i.e., E5, E6, E7, E8, E9, E10, and E11), which showed that GNS was strongly and negatively correlated with TGW, GA, GC, GL, and GW (Supplementary Table S7).

**Table 1:** Parental and population means, ranges, and broad sense heritabilities for TGW, GNS, GWS, and four grain morphometric parameters

Trait	Parental lines			RIL population			
	J411	Y8679	Delta (%)	Min	Max	Mean ± SD	$h_{\text{B}}^{2}$
TGW	47.56	64.95	36.54	42.36	65.23	55.73 ± 4.21	0.95
GNS	50.22	43.26	− 13.87	36.42	51.70	43.84 ± 2.95	0.90
GWS	2.41	2.74	13.69	2.00	2.89	2.47 ± 0.17	0.87
GA	17.49	22.80	30.39	16.68	22.53	19.82 ± 1.09	0.93
GC	16.89	20.05	18.67	16.95	19.89	18.41 ± 0.59	0.96
GL	6.54	7.95	21.66	6.53	7.88	7.22 ± 0.27	0.97
GW	3.37	3.62	7.58	3.14	3.71	3.47 ± 0.11	0.90

The traits include thousand grain weight (TGW), grain number per spike (GNS), grain weight per spike (GWS), grain surface area (GA), grain circumference (GC), grain length (GL), and grain width (GW). Delta refers to the difference between Y8679 and J411 phenotypes as a percentage of J411. Broad sense heritability based on a family mean basis was estimated across all evaluated environments for each trait. TGW and GNS were evaluated in 11 environments (from E1 to E11), whereas the four grain morphometric parameters were evaluated in seven environments (from E5 to E11)

### Identification of genomic regions harboring stable QTLs for TGW, GNS, GA, GC, GL, and GW

QTLs repeatedly detected in ≥ 3 individual environments and in the analysis of BLUPs were considered to be stable. According to this criterion, 34 stable QTLs for TGW, GNS, GA, GC, GL, and GW were mapped within eight genomic regions (Table 2; Supplementary Table S8). The corresponding physical intervals of these genomic regions in the Chinese Spring RefSeq v1.0 sequence are listed in Table 2 and Supplementary Table S9. Further 123 putative QTLs for TGW, GNS, GA, GC, GL, and GW that did not meet this criterion are listed in Supplementary Table S10. Of the 29 QTLs for GWS, two were detected in three (*QGws.cau*-*1B.3*) or four (*QGws.cau*-*4D.1*) environments, but not in the analysis of BLUPs (Supplementary Table S10).

**Table 2:** Eight genomic regions harboring stable QTLs for TGW, GNS, GA, GC, GL, and GW in the Y8679/J411 population

Chromosome	Interval (cM)^a^	Interval (Mb)^b^	Associated trait^c^	Included QTL^d^	Detected environment^e^	References
1B	0.0–7.7		GNS (Y)	***QGns.cau*** **-** ***1B.1***	E3/E7/E10/E11/C	Griffiths et al. (2015)
			GWS (Y)	***QGws.cau*** **-** ***1B.3***		
			GW (Y)	***QGw.cau*** **-** ***1B.1***	E6/E9/E11/C	
2B	117.0–129.6	691.78–727.21	GA (Y)	***QGa.cau*** **-** ***2B.1***; *QGa.cau*-*2B.2*	E6/E7/E8/C	
			GC (Y)	***QGc.cau*** **-** ***2B.1***; *QGc.cau*-*2B.2*	E5/E7/E8/E9/E11/C	
			GL (Y)	***QGl.cau*** **-** ***2B.2***; ***QGl.cau*** **-** ***2B.3***	E5/E6/E7/E8/E9/E10/E11/C	Sun et al. (2009)
3A	30.7–34.9	639.09–650.43	GNS (J)	***QGns.cau*** **-** ***3A.1***	E1/E7/E11/C	Cui et al. (2014)
3B	113.8–139.8	698.62–760.71	TGW (Y)	***QTgw.cau*** **-** ***3B.1***; ***QTgw.cau*** **-** ***3B.1***	E1/E4/E6/E8/E9/E11/C	Cui et al. (2014)
5B	6.0–28.4	35.29–395.63	TGW (Y)	***QTgw.cau*** **-** ***5B.2***; ***QTgw.cau*** **-** ***5B.3***	E2/E3/E5/E6/E7/C	Cui et al. (2014)
			GA (Y)	***QGa.cau*** **-** ***5B.2***	E6/E7/E11/C	
			GC (Y)	*QGc.cau*-*5B.1*; ***QGc.cau*** **-** ***5B.3***; *QGc.cau*-*5B.5*	E6/E7/E8/E9/E11/C	
6A	62.5–94.2	52.40–585.43	GL (Y)	***QGl.cau*** **-** ***5B.1***; ***QGl.cau*** **-** ***5B.3***; ***QGl.cau*** **-** ***5B.4***	E5/E6/E7/E8/E9/E10/E11/C	Cui et al. (2014)
			GNS (J)	***QGns.cau*** **-** ***6A.2***; ***QGns.cau*** **-** ***6A.3***; ***QGns.cau*** **-** ***6A.4***	E1/E2/E3/E4/E5/E6/E7/E8/E9/E10/E11/C	Jia et al. (2013)
			GNS (J)	***QGns.cau*** **-** ***6A.2***; ***QGns.cau*** **-** ***6A.3***; ***QGns.cau*** **-** ***6A.4***	E1/E2/E3/E4/E5/E6/E7/E8/E9/E10/E11/C	Jia et al. (2013)
			TGW (Y)	***QTgw.cau*** **-** ***6A.1***; ***QTgw.cau*** **-** ***6A.2***; ***QTgw.cau*** **-** ***6A.3***	E1/E2/E3/E4/E5/E6/E7/E9/E10/E11/C	Cui et al. (2014); Simmonds et al. (2014)
			GA (Y)	***QGa.cau*** **-** ***6A.1***; ***QGa.cau*** **-** ***6A.2***; ***QGa.cau*** **-** ***6A.3***	E5/E6/E7/E8/E9/E10/E11/C	
			GC (Y)	***QGc.cau*** **-** ***6A.2***; *QGc.cau*-*6A.3*; ***QGc.cau*** **-** ***6A.4***	E5/E6/E7/E8/E9/E11/C	
			GL (Y)	*QGl.cau*-*6A.1*; ***QGl.cau*** **-** ***6A.2***	E5/E6/E7/E8/E9/E11/C	
			GW (Y)	*QGw.cau*-*6A.1*; *QGw.cau*-*6A.2*; ***QGw.cau*** **-** ***6A.3***	E5/E6/E7/E8/E9/E11/C	Cui et al. (2014)
6B	1.3–31.9		GL (Y)	***QGl.cau*** **-** ***6B.2***; ***QGl.cau*** **-** ***6B.3***	E5/E6/E9/E11/C	
7A	53.9–85.2	76.98–275.92	GNS (J)	***QGns.cau*** **-** ***7A.2***; ***QGns.cau*** **-** ***7A.3***	E3/E5/E6/E7/E9/E10/C	Kumar et al. (2007)
			GL (Y)	***QGl.cau*** **-** ***7A.1***	E5/E7/E11/C	

^a^Additional details regarding the SNP markers within each QTL region can be found in Zhai et al. (2016)

^b^The corresponding physical intervals (Mb) of the QTL regions on chromosomes 2B, 3A, 3B, 5B, 6A or 7A were obtained by blasting the flanking sequences of SNP markers to the Chinese Spring RefSeq v1.0 sequence (Supplementary Table S9)

^c^The traits include thousand grain weight (TGW), grain number per spike (GNS), grain surface area (GA), grain circumference (GC), grain length (GL) and grain width (GW). The letters within the brackets indicate the origin of the increasing alleles, with ‘Y’ and ‘J’ representing Y8679 and J411, respectively

^d^QTLs shown in bold are stable QTLs that were detected in ≥ 3 individual environments and in the analysis of BLUPs

^e^C indicates the combined QTL analysis based on BLUP values

**Fig. 1:**
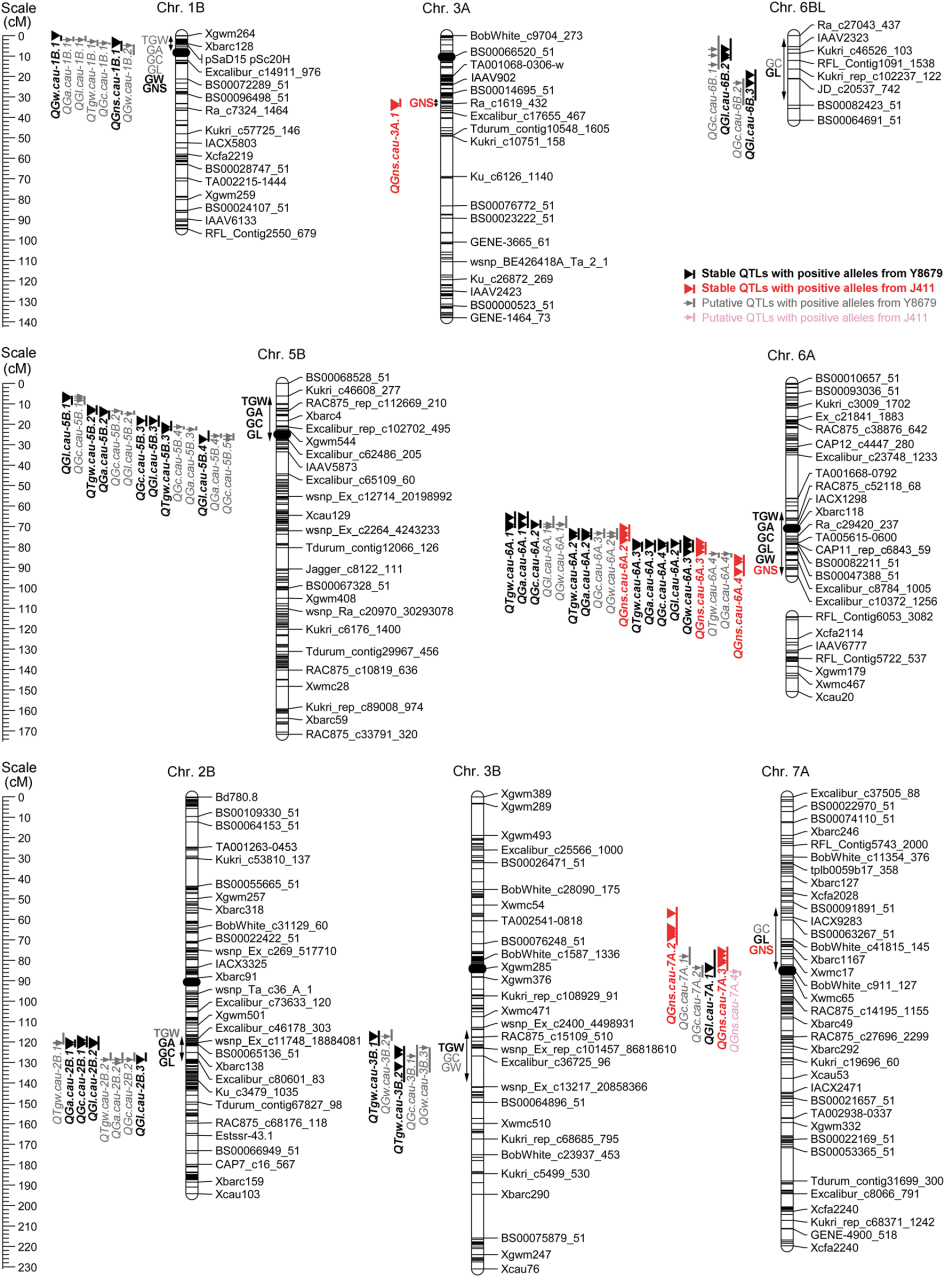
Chromosomal locations of eight genomic regions associated with TGW, GNS, and four grain morphometric parameters in the Y8679/J411 population. Three centiMorgan (cM) scales are shown on the left. Information regarding the omitted SNP and SSR markers (represented as horizontal black lines) can be accessed in Zhai et al. (2016). Solid black ellipses indicate the centromeres. Double-headed arrows specify the interval of a genomic region harboring QTLs or QTL clusters. Vertical bars represent the LOD-2 confidence intervals of each QTL, with triangles or arrows indicating the QTL peaks in individual environments. Stable QTLs are shown in bold black or bold red, with superior alleles coming from Y8679 or J411, respectively. Putative QTLs are displayed in gray and pink, with Y8679 or J411 contributing the increasing alleles, respectively

**Fig. 2:**
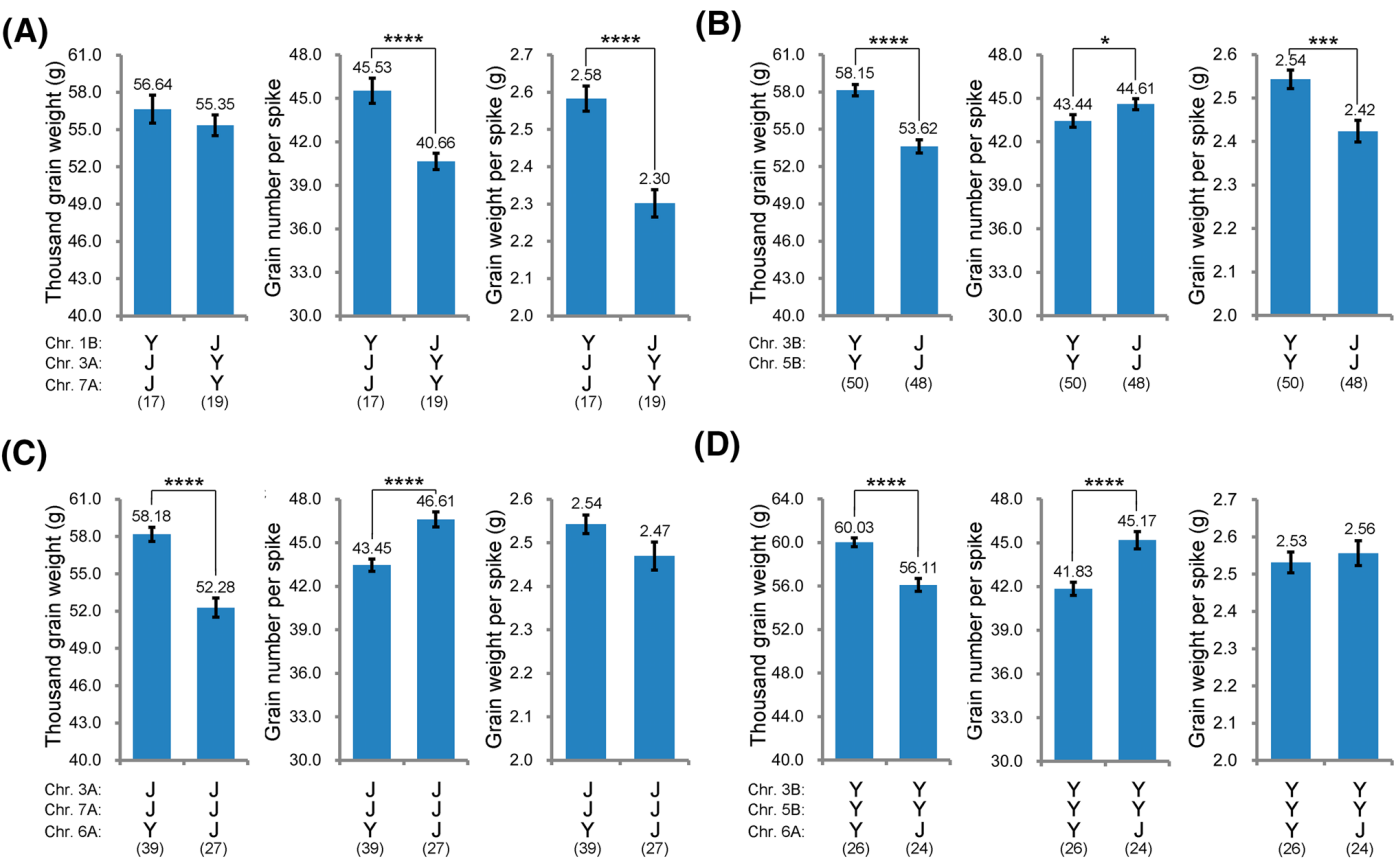
Pyramiding effects of several stable QTL regions on chromosomes 1B, 3A, 3B, 5B, 6A, or 7A. The phenotypic data used here were the BLUP estimates of each recombinant inbred line (RIL) across 11 environments. The genotypic data used here were collected from the allelic information at specific markers within the stable QTL regions as follows: *pSc20H* (Chr. 1B), *Ra_c1619_432* (Chr. 3A), *RAC875_c15109_510* (Chr. 3B), *Excalibur_rep_c102702_495* (Chr. 5B), *Ra_c29420_237* (Chr. 6A), and *BobWhite_c41815_145* (Chr. 7A). ‘Y’ and ‘J’ indicate alleles contributed by Y8679 and J411, respectively. For a specific pyramiding pattern, the number of RILs is shown in the bracket, and the mean value of these RILs (± SE) is shown in a histogram. *, ***, and **** indicate significant differences at the 0.05, 0.001, and 0.0001 levels (Student’s *t* test), respectively

The stable QTL regions on chromosomes 1B, 3A, and 7A were found to have an effect on GNS, but with no significant effect on TGW (Fig. 1; Table 2). The region on chromosome 1B co-localized with the 1RS/1BL translocation, which is present in Y8679 and has a strong positive effect on spikelet number per spike (Zhai et al. 2016). *QGns.cau*-*1B.1* was the only GNS QTL at which Y8679 contributed the increasing allele. In detected environments, *QGns.cau*-*1B.1* explained 8.49–15.25% of the total variation of GNS. The region on chromosome 3A contained another stable QTL for GNS (*QGns.cau*-*3A.1*) with a relatively minor effect. The region on chromosome 7A contained two adjacent major GNS QTLs (*QGns.cau*-*7A.2* and *QGns.cau*-*7A.3*) that together explained 29.63% of the observed variation of GNS. For both regions on chromosomes 3A and 7A, the alleles from J411 were found to have significant positive effects on GNS. The RILs carrying positive alleles at all three of the stable QTL regions for GNS (YJJ) exhibited significantly higher GNS (by 4.87 grains, *P* < 0.0001) and GWS (by 0.28 g, *P* < 0.0001) (Fig. 2a) than those possessing the three opposite alleles (JYY). The two groups did not differ significantly (*P* = 0.36) in TGW.

The stable QTL regions on chromosomes 3B and 5B mainly influenced TGW, with the superior alleles coming from Y8679 (Fig. 1; Table 2). In the analysis of BLUPs, *QTgw.cau*-*3B.1* and *QTgw.cau*-*3B.2* explained 7.35 and 6.44% of the variation observed for TGW, respectively. A QTL for GW (*QGw.cau*-*3B.2*) coincided with *QTgw.cau*-*3B.1*, suggesting possible pleiotropy at this locus. The region on chromosome 5B covered two stable QTLs for TGW, i.e., *QTgw.cau*-*5B.2* and *QTgw.cau*-*5B.3*, which together explained 12.36% of the total variation of TGW. This region also possessed stable QTLs for GA (*QGa.cau*-*5B.2*), GC (*QGc.cau*-*5B.3*), and GL (*QGl.cau*-*5B.3* and *QGl.cau*-*5B.4*), indicating that the positive effect on TGW was most likely conferred by increasing GL. The RILs carrying positive alleles in both the 3B and 5B regions (YY) had higher TGW (by 4.53 g, *P* < 0.0001) and GWS (by 0.12 g, *P* < 0.001) and slightly lower GNS (by 0.12 grains, *P* < 0.05) than those possessing the two opposite alleles (JJ) (Fig. 2b).

**Fig. 3:**
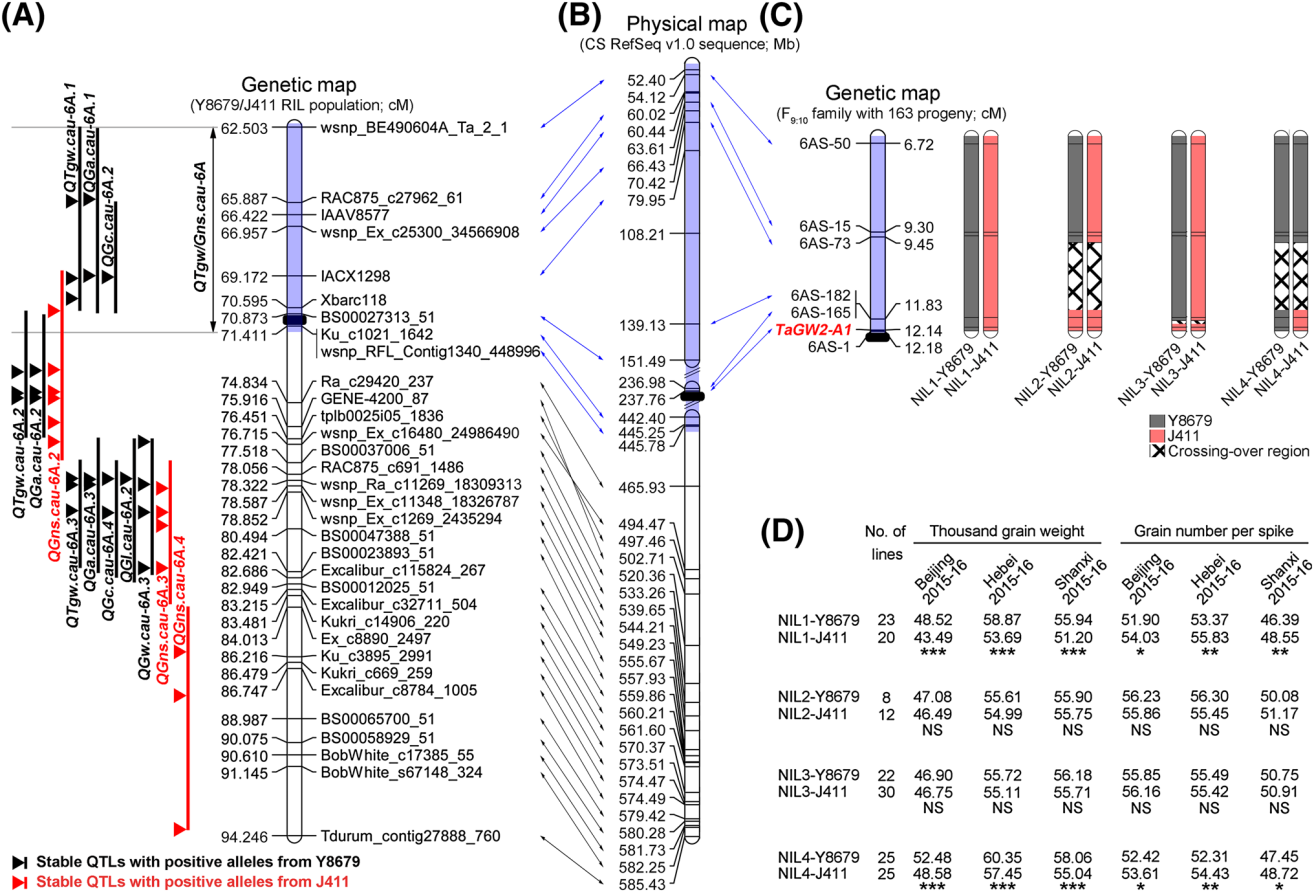
Fine mapping of *QTgw/Gns.cau*-*6A*. **a** Distributions of major QTLs for TGW, GNS, and four grain morphometric parameters within the stable QTL region on chromosome 6A. The interval colored in light blue represents *QTgw/Gns.cau*-*6A*, which possessed whole *QTgw.cau*-*6A.1* and partial *QGns.cau*-*6A.2*. Descriptions regarding the QTL confidence intervals and the peak regions are consistent with those introduced in Fig. 1. The solid black ellipse indicates the centromere. **b** Corresponding physical interval of the stable QTL region on chromosome 6A in the Chinese Spring RefSeq v1.0 sequence. The solid black ellipse indicates the centromere. **c** Graphical genotypes of four NIL pairs (derived from RIL186) with overlapping recombinant segments. The solid black ellipse indicates the centromere. **d** Performance of the members of the four NIL pairs in three field trials. *, **, and *** indicate significant differences at the 0.05, 0.01, and 0.001 levels (Student’s *t* test), respectively

In the stable QTL region on chromosome 6A, the allele from Y8679 increased TGW but decreased GNS (Fig. 3a; Table 2). This region contained three major QTLs for TGW, i.e., *QTgw.cau*-*6A.1*, *QTgw.cau*-*6A.2,* and *QTgw.cau*-*6A.3*, which together explained 49.20% of the total variation of TGW. Moreover, several major QTLs for GA (*QGa.cau*-*6A.1*, *QGa.cau*-*6A.2,* and *QGa.cau*-*6A.3*) and GC (*QGc.cau*-*6A.2* and *QGc.cau*-*6A.4*) were also detected in this region. Together, these QTLs explained 50.14 and 27.08% of the total variation of GA and GC, respectively. Y8679 contributed increasing alleles for all of these loci. Two major QTLs for GNS (*QGns.cau*-*6A.2* and *QGns.cau*-*6A.3*) with superior alleles coming from J411 were also detected in this region. Together, these QTLs explained 38.60% of the observed GNS variation. Even in the presence of high-GNS alleles on chromosomes 3A and 7A or high-TGW alleles on chromosomes 3B and 5B, the RILs with Y8679 allele at the 6A region had higher TGW (by 3.92–5.90 g, *P* < 0.0001) and lower GNS (by 3.16–3.24 grains, *P* < 0.0001) than those possessing J411 allele (Fig. 2c, d).

For GL, there were also additional stable QTLs in regions on chromosomes 2B and 6B, but these QTLs did not significantly influence TGW (Figs. 1, 2). The region on chromosome 2B harbored the two most stable QTLs for GL, i.e., *QGl.cau*-*2B.2* and *QGl.cau*-*2B.3*, which were found in nearly all evaluated environments (Supplementary Table S8). These two loci together explained 19.02% of the detected GL variation. *QGl.cau*-*2B.2* co-localized with two stable QTLs for GA (*QGa.cau*-*2B.1*) and GC (*QGc.cau*-*2B.1*), which explained 4.92 and 8.81% of the total variation of GA and GC, respectively. The region on chromosome 6B contained two QTLs for GL, i.e., *QGl.cau*-*6B.2* and *QGl.cau*-*6B.3*, which together explained 13.81% of the observed GL variation.

### Use of NILs to verify and precisely map a QTL on chromosome 6A

RIL186 was selected from the population, because this RIL exhibited residual heterozygosity at locus *Xbarc118*. Two of its selfed progeny, which possessed alternative haplotypes and were homozygous, were genotyped using the Affymetrix wheat 660 K SNP array (http://wheat.pw.usda.gov/ggpages/topics/

Wheat660_SNP_array_developed_by_CAAS.pdf). The results showed that they were 99.82% similar, only differing in 1094 SNPs (Supplementary Table S11). Of these 1094 polymorphic SNPs, 895 were located in an interval between 28.22 and 445.78 Mb on chromosome 6A. The flanking sequences of these 895 SNP markers and their physical locations in the Chinese Spring RefSeq v1.0 sequence are listed in Supplementary Table S12. Considering that the corresponding physical interval of the 6A region spanned from 52.40 to 585.43 Mb, we chose to focus on the region from 52.40 to 445.78 Mb (Table 2), i.e., an 8.908 cM interval between *wsnp_BE490604A_Ta_2_1* and *wsnp_RFL_Contig1340_448996* and designated as *QTgw/Gns.cau*-*6A*, for the development of NILs (Fig. 3a, b).

Fourteen co-dominant polymorphic SSR markers (Supplementary Table S6) were developed using a collinearity-based strategy and used to genotype the F_9:10_ family with 163 individuals derived from RIL186. The resultant genetic linkage map spanned 12.18 cM in length, covering an interval from 31.39 to 237.76 Mb in the Chinese Spring RefSeq v1.0 sequence (Supplementary Table S13). Based on genotype of these markers, four sets of NIL pairs (NIL1, NIL2, NIL3, and NIL4) with overlapping recombinant segments were developed (Fig. 3c). The first NIL pair, with no recombination in the concern region, was used to verify the effects of *QTgw/Gns.cau*-*6A*.

**Fig. 4:**
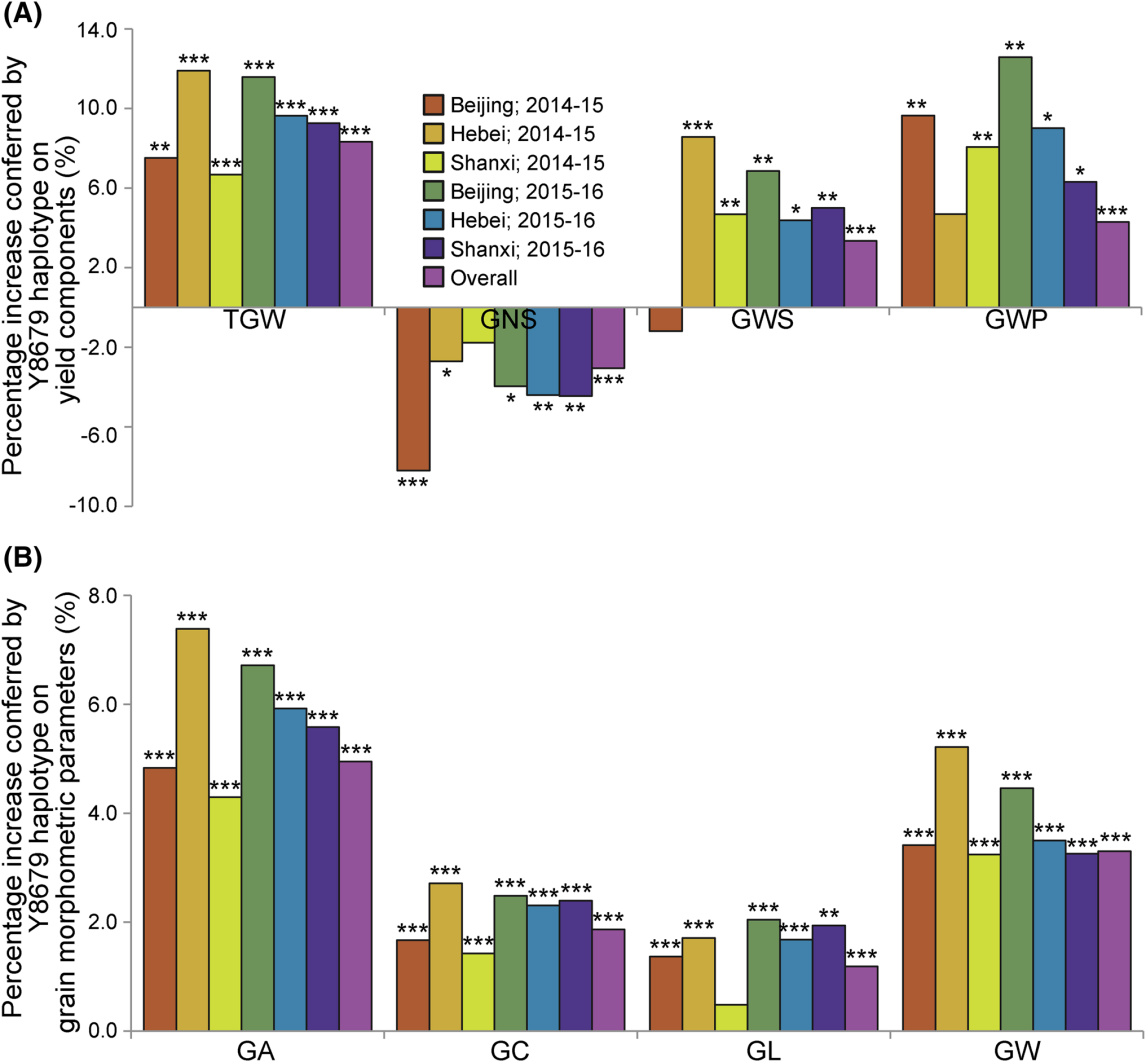
Phenotypic comparisons of NIL1-Y8679 and NIL1-J411 in six field trials. Percentage increase conferred by the Y8679 haplotype on **a** yield components and **b** grain morphometric parameters. Significant differences are indicated by *P < 0.05, **P < 0.01, and ***P < 0.001 (Student’s *t* test). ‘Overall’ represents the BLUP values estimated across six evaluated environments

Across six replicated field trials, the TGW of NIL1-Y8679 (with Y8679 haplotype) was from 6.68 to 11.90% (mean 8.33%, *P* < 0.001) higher than that of NIL1-J411 (Fig. 4; Supplementary Table S14). This variation in TGW was associated with average differences of 4.95, 1.87, 1.19, and 3.30% (*P* < 0.001) in GA, GC, GL, and GW, respectively, with NIL1-Y8679 having the higher values. A difference in GNS (mean 3.05%, *P* < 0.001) was also observed, with NIL1-Y8679 exhibiting the lower value. This effect varied across six trials with NIL1-Y8679 associated with significant GNS reductions (*P* < 0.05) from 2.71 to 8.20% in five of them. Overall, NIL1-Y8679 had 3.35% higher GWS (*P* < 0.001) and 4.29% higher GWP (*P* < 0.001) than NIL1-J411. In most trials, no significant differences were observed for tiller number (Supplementary Table S14). Collectively, these results demonstrate that the Y8679 haplotype in *QTgw/Gns.cau*-*6A* has a consistent positive effect on GWS and GWP, which is conferred by a large positive effect on TGW, but counterbalanced by a small negative effect on GNS.

Subsequently, we used NIL2, NIL3, and NIL4 to map the high-TGW, low-GNS region into a smaller genetic interval. When these NIL pairs were evaluated for TGW and GNS in three field trials, significant differences in TGW and GNS were found only between the members of the NIL4 pair (Fig. 3d), but not between the members of the NIL2 or NIL3 pairs. Based on these results, *QTgw/Gns.cau*-*6A* was narrowed to an interval of 208.80 Mb, which is flanked by marker *6AS*-*165* (236.98 Mb) and the distal margin of the region of interest (445.78 Mb; Supplementary Table S12). This represents a genetic interval shorter than 0.538 cM on the Y8679/J411 map, a segment flanked by *BS00027313_51* (70.873 cM; 151.49 Mb) and *wsnp_RFL_Contig1340_448996* (71.411 cM; 445.25 Mb) (Fig. 3a).

To estimate the additive and dominance effects of *QTgw/Gns.cau*-*6A*, we evaluated the 163 F_10:11_ families for TGW, GNS, and GWS in one field trial (Beijing, 2014–15 crop season) in three replicates. Eighty segregating families and 36–41 homozygous families with alternative haplotypes across the interval from *6AS*-*165* to *6AS*-*1* were used for variation analysis. The Y8679 haplotype of *QTgw/Gns.cau*-*6A* showed overdominance (*d/a* = 1.76) for higher TGW, with the mean values of segregating families (43.51 g) and Y8679-type homozygous families (43.75 g) being significantly higher (*P* < 0.001) than that of J411-type homozygous families (39.78 g). Likewisely, the J411 haplotype of *QTgw/Gns.cau*-*6A* exhibited overdominance (*d/a* = 2.16) for higher GNS, with the segregating families and J411-type homozygous families bearing 2.80 and 2.69 more grains (*P* < 0.001) than the Y8679-type homozygous families, respectively. We also observed an overdominance effect (*d/a* = 11.04) of *QTgw/Gns.cau*-*6A* on spike yield, with the mean GWS values of segregating families being 0.11–0.16 g higher (*P* < 0.01) than those of the two types of homozygous families (Table 3).

**Table 3:** Estimation of the additive and dominance effects of *QTgw/Gns.cau*-*6A* on TGW, GNS, and GWS using 163 F_10:11_ families

Families	TGW	GNS	GWS
Y8679-type homozygous families	43.75 ± 0.50^A^	41.36 ± 0.59^B^	1.82 ± 0.04^B^
Segregating families	43.51 ± 0.28^A^	44.16 ± 0.33^A^	1.93 ± 0.02^A^
J411-type homozygous families	39.78 ± 0.30^B^	44.05 ± 0.35^A^	1.77 ± 0.02^B^
*a*	1.98	1.35	0.02
*d*	3.49	2.91	0.26
*d/a*	1.76	2.16	11.04

The 163 F_10:11_ families derived from RIL186 were evaluated at Beijing during the 2014–15 crop season with three replicates. Based on the genotypes of *6AS*-*165* and *6AS*-*1*, 80 segregating families, 41 Y8679-type homozygous families and 36 J411-type homozygous families were selected and used for phenotypic variation analysis. *a* and *d* indicate the additive and dominance effects, respectively. *d/a* indicates the degree of dominance. Multiple comparison was based on LSD method. Different letters (^A^ and ^B^) are used to indicate the means (± SE) that significantly differ (*P* < 0.01)

### Isolation of candidate gene *TaGW2*-*A1*

**Fig. 5:**
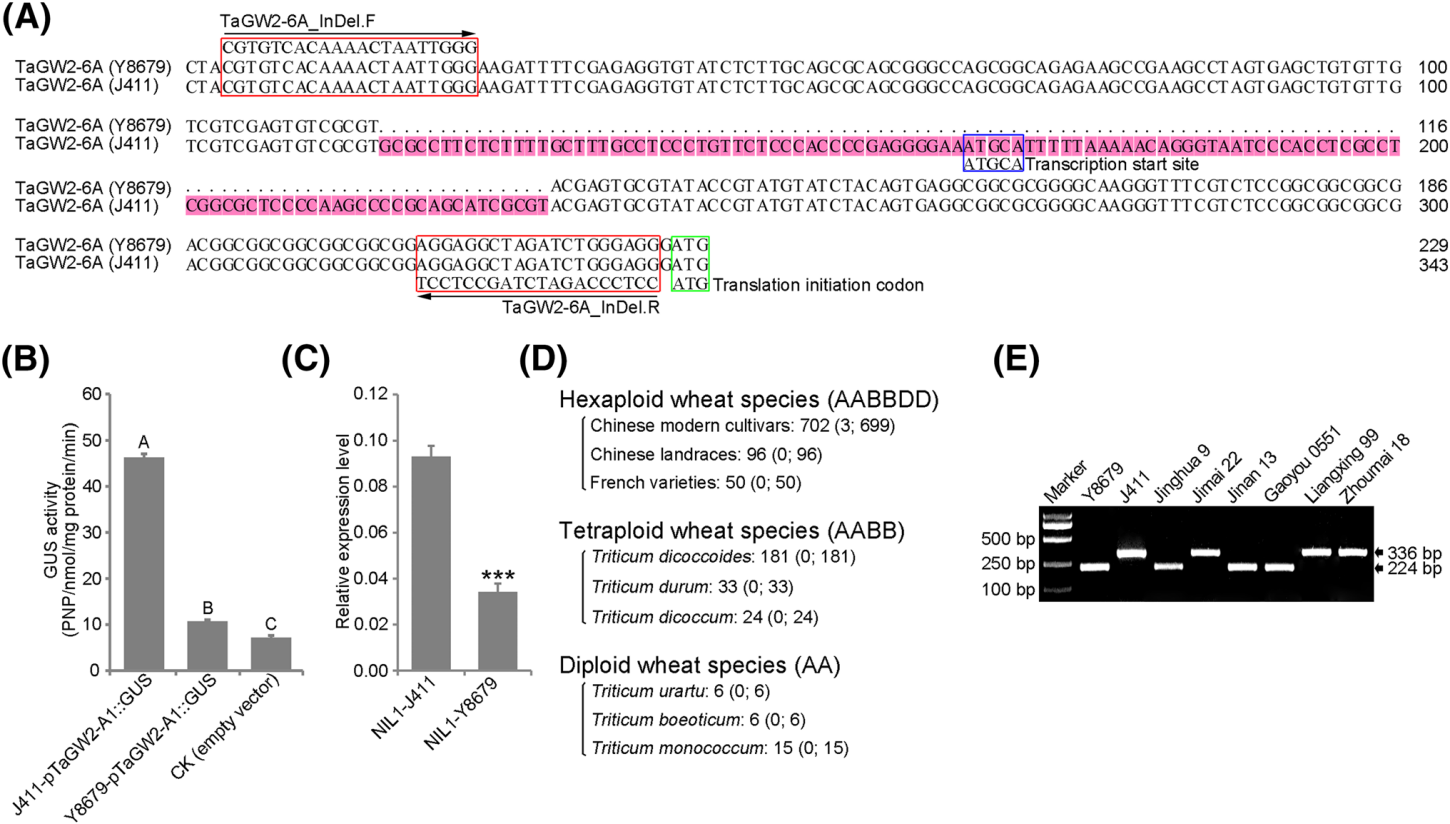
Isolation of a novel and rare allele of *TaGW2*-*A1*. **a** 114-bp Insertion/Deletion (InDel) detected in the promoter region. Red rectangles specify the forward and reverse primers of TaGW2-A1_InDel. Blue and green rectangles indicate the transcription start site and the predicted translation initiation codon, respectively. **b** Comparison of the GUS expression level in different promoter-GUS constructs. Superscripts A, B, and C indicate the means (± SE) that significantly differ at the 0.01 level (LSD). **c** Relative expression levels of *TaGW2*-*A1* in immature grains (11 dap) of the NIL1 pair, with *Actin* used as the endogenous control. The results of three biological replications showed similar trends. The values represent the means (± SE) of three biological replicates. *** indicates significance at the 0.001 level (Student’s *t* test). **d** Frequency of the 114-bp deletion among 1113 wheat accessions with varying ploidy. The two numbers (from left to right) within the brackets indicate the number of accessions amplifying the 224-bp fragment and the 336-bp fragment, respectively. **e** PCR products of TaGW2-A1_InDel in several hexaploid wheat accessions

*TaGW2*-*A1*, a wheat homolog of rice *OsGW2* (Song et al. 2007), was previously reported to negatively affect TGW in wheat (Du et al. 2016; Simmonds et al. 2016; Yang et al. 2012). It was mapped to the short arm of chromosome 6A, near the centromere (Su et al. 2011). As this position corresponds with that of *QTgw/Gns.cau*-*6A*, we obtained the complete coding sequence and the promoter sequence (about 2.0 kb upstream from ATG) for *TaGW2*-*A1* from Y8679 to J411, and analyzed their polymorphisms. No differences were observed in the coding sequence, but a 114-bp Insertion/Deletion (InDel) was detected in the promoter region (− 230 to − 117-bp upstream from ATG; Fig. 5a). No other variations were detected in the rest of the promoter sequence. The allele from Y8679 has the 114-bp deletion as compared to those of J411 and Chinese Spring (Supplementary Fig. S6). The primer pair TaGW2-A1_InDel targeting this InDel was used to genotype the F_9:10_ family with 163 progeny derived from RIL186. *TaGW2*-*A1* was mapped to chromosome 6A near marker *6AS*-*1* (Fig. 3c), within the finely mapped interval of *QTgw/Gns.cau*-*6A*.

We obtained the full-length cDNA of *TaGW2*-*A1* by 5′-RACE and 3′-RACE using RNA isolated from 7-day-old seedlings of J411. The obtained full-length cDNA was 1675 bp in length, including a 176-bp 5′ untranslated region (UTR), a 1275-bp ORF, a 3-bp stop codon (TAA), and a 221-bp 3′UTR. The transcription start site (− 176-bp upstream from ATG) was located within the 114-bp InDel (Fig. 5a). We fused the *TaGW2*-*A1* promoter sequences from Y8679 (1121 bp) and J411 (1235 bp) with the GUS reporter gene (Supplementary Fig. S3), respectively, to assess the effects of the 114-bp InDel on promoter activity. The GUS activity conferred by Y8679-pTaGW2-A1::GUS was 76.83% lower (*P* < 0.001) than that by J411-pTaGW2-A1::GUS, but still 48.96% higher (*P* < 0.01) than that detected for the empty vector control (Fig. 5b). In the immature grains (11 days after pollination), the expression level of *TaGW2*-*A1* in NIL1-Y8679 was 63.27% lower (*P* < 0.001) than that in NIL1-J411 (Fig. 5c). Taken together, we propose that the 114-bp deletion reduces the promoter activity and expression of *TaGW2*-*A1*.

To further explore implications of our findings on wheat breeding, primer pair TaGW2-A1_InDel was used to amplify 848 hexaploid wheat accessions, including 798 Chinese varieties (96 landraces and 702 modern cultivars) and 50 French varieties (Fig. 5d; Supplementary Table S1). Nearly all accessions (99.6%) amplified the wild-type 336-bp J411 fragment, whereas only three (Jinan 13, Jinghua 9 and Gaoyou 0551) amplified the mutant type 222-bp Y8679 fragment (Fig. 5e; Supplementary Fig. S7). Thus, at least within the panel evaluated, the favorable allele of *TaGW2*-*A1* is very rare. Moreover, 238 tetraploid and 27 diploid wheat species were also screened for the 114-bp deletion (Fig. 5d; Supplementary Tables 2 and 3), but none of them carried the 224-bp fragment like Y8679, again suggesting a rare mutation event leading to the 114-bp deletion.

## Discussion

### Five genomic regions exhibit negligible TGW-GNS trade-offs

TGW and GNS are two primary yet negatively correlated determinants of spike yield in wheat (Jia et al. 2013; Shukla et al. 2015). Information about trade-offs between the two traits can help to improve the efficiency of wheat breeding. Here, we report five genomic regions on chromosomes 1B, 3A, 3B, 5B, or 7A exhibiting negligible TGW-GNS trade-offs. These represent attractive targets for marker-assisted selection to enhance grain size and/or grain number. The region on chromosome 1B can be attributed to the 1RS/1BL translocation (carried by Y8679). It increased GNS, corresponding to our previous finding that 1RS/1BL translocation enhanced spikelet number per spike across all evaluated trials (Zhai et al. 2016). In the regions on chromosomes 3A and 7A, alleles from J411 increased the grain number without significantly reducing grain weight. The 3A region is probably within the deletion bin 3AL5-0.78-1.00, which has previously been reported to harbor QTLs for grain yield, GNS, spikelet number per spike, test weight, TGW, and heading time (Ali et al. 2011; Cui et al. 2014; Huang et al. 2004; Zhai et al. 2016). Similarly, the region detected on chromosome 7A has previously been reported to affect GNS, spikelet number per spike and grain yield (Kumar et al. 2007; Ma et al. 2007; Reif et al. 2011; Wu et al. 2012). In the regions on chromosomes 3B and 5B, alleles from Y8679 increased TGW without significantly reducing GNS. These two regions coincide with several previously reported QTLs for TGW and grain yield (Cui et al. 2014; Reif et al. 2011; Sun et al. 2009). Notably, the region on chromosome 3B has also been shown to enlarge grain size through enhancing grain filling rate in the Heshangmai/Y8679 population, which shares a common parent with the population used in this study (Wang et al. 2009).

### Analysis of the QTLs on chromosome 6A provides new insight into TGW-GNS trade-off

In recent years, evidence is accumulating that robust QTLs for TGW (Cui et al. 2016; Mir et al. 2012; Snape et al. 2007; Zanke et al. 2015) and GNS (Jia et al. 2013; Yuan et al. 2012) exist in the pericentromeric region of chromosome 6A. Here, we detected stable major QTLs for TGW (*QTgw.cau*-*6A.1*, *QTgw.cau*-*6A.2* and *QTgw.cau*-*6A.3*) and GNS (*QGns.cau*-*6A.2,* and *QGns.cau*-*6A.3*) in this region. The two QTL clusters on chromosome 6A for TGW and GNS were both linked in the coupling phase, but with superior alleles coming from opposite parents. Hence, selection for the higher TGW haplotype would inevitably be accompanied by a reduction of GNS, and vice versa. This is a typical selection trade-off problem for higher TGW versus higher GNS. Importantly, by evaluating a pair of NILs in several field trials, we demonstrated that *QTgw/Gns.cau*-*6A* (covering whole *QTgw.cau*-*6A.1* and partial *QGns.cau*-*6A.2*) affected both TGW and GNS in an opposite manner. Through further mapping with another three pairs of NILs, we narrowed *QTgw/Gns.cau*-*6A* to a small genetic interval shorter than 0.538 cM. Consequently, *QTgw/Gns.cau*-*6A* and the NIL pairs developed here may be a perfect target and good materials, respectively, for further studying the molecular genetic basis of TGW–GNS trade-off in common wheat.

From a practical point of view, it is worthy to point out that when pyramiding the QTLs identified here, it seems advisable to select the Y8679 haplotype in the presence of major QTLs for GNS on chromosomes 3A and 7A (Fig. 2c), or the J411 haplotype in the presence of major QTLs for TGW on chromosomes 3B and 7B (Fig. 2d).

### A novel mutation of *TaGW2*-*A1* is associated with increased TGW in wheat

In rice, *OsGW2* encodes a RING-type protein with E3 ubiquitin ligase activity that negatively regulates the grain weight and width (Song et al. 2007). Its wheat homolog on the short arm of chromosome 6A (*TaGW2*-*A1*) locates in a region near the centromere (Su et al. 2011), in which major QTLs for TGW have been mapped (Cui et al. 2016; Simmonds et al. 2014; Zanke et al. 2015). Several reports (Su et al. 2011; Zhang et al. 2013, Jaiswal et al. 2015) have focused on the roles of individual SNPs in the promoter region, but validations of their associations with TGW are still needed. Two mutations in the coding sequence have been reported to be associated with TGW: a frame-shift mutation in exon 8 (Du et al. 2016; Yang et al. 2012) and a splice acceptor site mutation in exon 5 (Simmonds et al. 2016). The effects of these coding sequence mutations have been tested in backcross derived isogenic lines, providing sound evidence that *TaGW2*-*A1,* indeed, influences TGW in tetraploid and hexaploid wheat.

In the present study, we located *TaGW2*-*A1* within the finely mapped interval of *QTgw/Gns.cau*-*6A*. Furthermore, a novel mutation of *TaGW2*-*A1* with a 114-bp deletion in the promoter was isolated from the high-TGW parent Y8679. Deletion of the wild-type transcription start site reduced promoter activity and decreased expression of *TaGW2*-*A1*, which might, in turn, enhance TGW according to the previous finding that *TaGW2*-*A1* is a negative regulator of grain weight (Du et al. 2016; Simmonds et al. 2016; Yang et al. 2012). In a germplasm survey of more than 800 hexaploid wheat accessions, the 114-bp deletion was detected only in Y8679 and three other cultivars: Jinan 13, Jinghua 9, and Gaoyou 0551 (Fig. 5e). These four cultivars could be useful as donors for higher TGW in wheat breeding programs. Due to the incomplete pedigree information (Supplementary Table S15), no clear relationship was observed in these accessions. Furthermore, this novel allele was not detected in tetraploid and diploid wheat species that were involved in the formation of hexaploid wheat (IWGSC 2014). Together, these data point to the possibility that the novel *TaGW2*-*A1* allele identified here may arise during or after the hexaploidization event that yielded hexaploid wheat.

### *QTgw/Gns.cau*-*6A* shows possible overdominance effect on spike yield

Heterosis has been a main contributor to yield increase in many cereal crops (Fu et al. 2014). Despite extensive efforts in hybrid wheat breeding, mechanisms of wheat heterosis are largely unknown (Ni et al. 2013). Here, we provide the first example of overdominance effect on spike yield that conferred by a single locus in wheat. Data from a single year trial (Beijing; 2014–15) suggest that the combination of the Y8679 haplotype and the J411 haplotype of *QTgw/Gns.cau*-*6A* in the heterozygotes will most likely lead to an overdominance effect on GWS. Using the NILs developed for *QTgw/Gns.cau*-*6A*, the consistency of this overdominance effect across environments will be further investigated in the future.

Previously, Bednarek et al. (2012) found that *TaGW2* knockdown through RNAi caused no significant alternation in GNS or in the number of spikelets per spike. Similarly, Hong et al. (2014) also found that *TaGW2*-RNAi had no significant impact on grain number per plant, and Simmonds et al. (2016) found that a G-to-A transition in the splice acceptor site of exon 5, which leads to mis-splicing in *TaGW2*-*A1*, had no significant impact on GNS or on the number of spikelets per spike. Thus, we tend to believe that the overdominance effect of *QTgw/Gns.cau*-*6A* on GWS was most likely caused by two or more different dominant genes linked in repulsion phase, with *TaGW2*-*A1* for enlarged grain size, while the other for increased grain number. Dissection of *QTgw/Gns.cau*-*6A* into two distinct QTLs for TGW and GNS, respectively, may eliminate linkage drag, but it will be a challenging task, since *QTgw/Gns.cau*-*6A* is located near the centromere. Considering the low recombination frequency of the pericentromeric region, utilization of the overdominance effect on GWS in the form of heterozygotes seems to be a feasible way to combine higher TGW and higher GNS phenotypes.

#### **Author contribution statement**

ZN and QS conceived the project; SX developed the RIL population; HZ, ZF, and JL performed phenotyping of the RIL population under 11 environments; XD, YS, HZ, and LS performed cloning and expressional analyses of *TaGW2*-*A1*. HZ and ZQ performed marker development of the QTL region of interest; HZ developed the near isogenic lines; HZ, LL, and JL performed phenotyping of the near isogenic lines; HP, ZH, YY, and MX assisted in revising the manuscript; HZ and ZN analyzed experimental results and wrote the manuscript.

## Electronic supplementary material

Below is the link to the electronic supplementary material.

Supplementary material 1 (DOC 11524 kb)

Supplementary material 2 (XLS 589 kb)
