# Supplementary material for: A novel allele of TaGW2-A1 is located in a finely mapped QTL that increases grain weight but decreases grain number in wheat (Triticum aestivum L.)
Source: Theor Appl Genet. 2017 Nov 17;131(3):539–53. doi: 10.1007/s00122-017-3017-y (PMC5814529; doi:10.1007/s00122-017-3017-y)
Supplement: Supplementary file 1 — Supplementary material 1 (DOC 11524 kb) [file 122_2017_3017_MOESM1_ESM.doc]

**Supplementary Figures S1-S7**

**A Novel Allele of *TaGW2-A1* Is Located in a Finely Mapped QTL that Increases Grain Weight but Decreases Grain Number in Wheat (*Triticum aestivum* L.)**

Huijie Zhai^1, 2^, Zhiyu Feng^1, 2^, Xiaofen Du^1, 2^, Yane Song^1, 2^, Xinye Liu^1, 2^, Zhongqi Qi^1, 2^, Long Song^1, 2^, Jiang Li^1, 2^, Linghong Li^1, 2^, Shihe Xiao^3^, Mingming Xin^1, 2^, Zhaorong Hu^1, 2^, Yingyin Yao^1, 2^, Huiru Peng^1, 2^, Qixin Sun^1, 2^ and Zhongfu Ni^1, 2^

^1^State Key Laboratory for Agrobiotechnology, Key Laboratory of Crop Heterosis and Utilization, Beijing Key Laboratory of Crop Genetic Improvement, China Agricultural University, Beijing 100193, China. ^2^National Plant Gene Research Centre, Beijing 100193, China. ^3^Institute of Crop Science, Chinese Academy of Agricultural Sciences, Beijing 100081, China

**Author for correspondence:** Zhongfu Ni; [nizf@cau.edu.cn](mailto:nizf@cau.edu.cn); +086 010 62734421


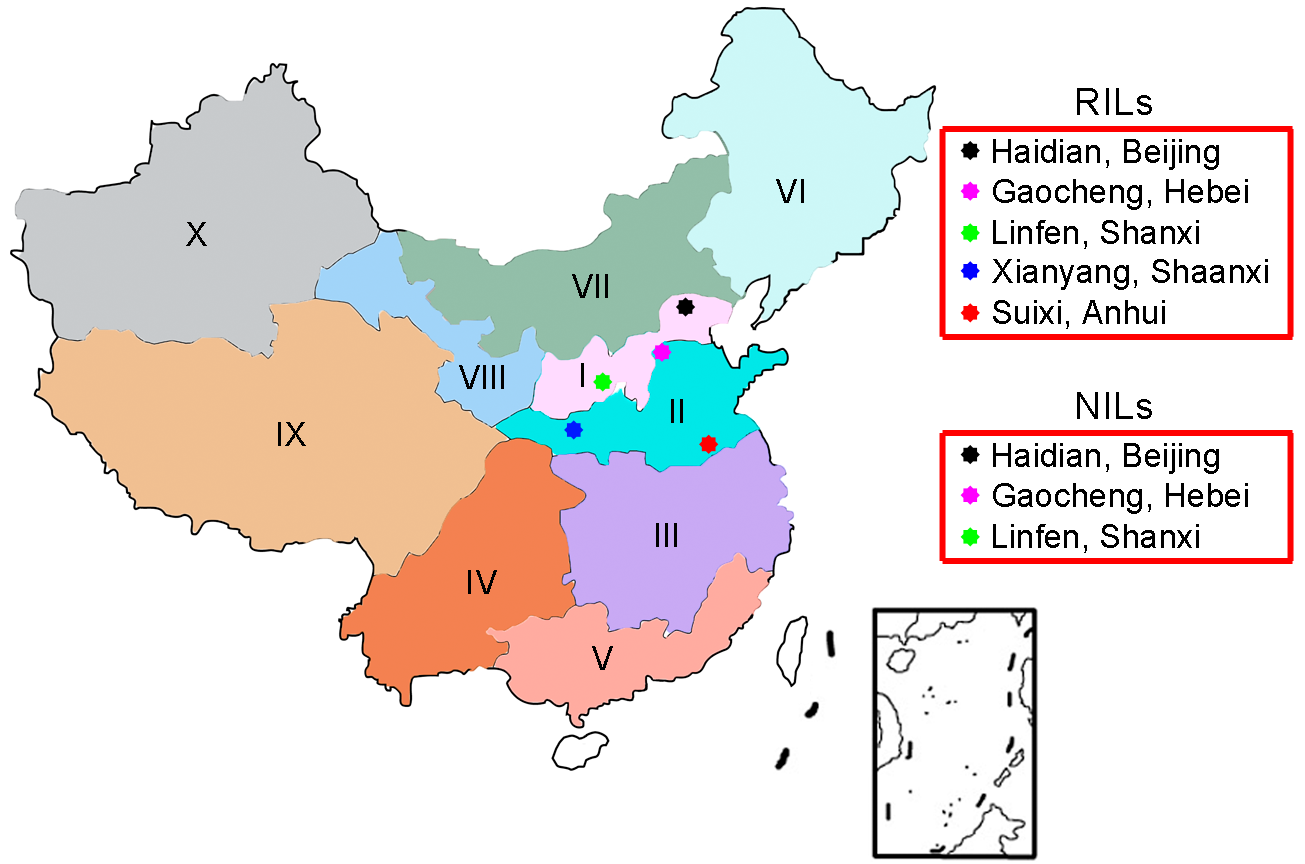


**Fig. S1** Location information for the field trials. Five and three locations were selected for evaluation of the recombinant inbred lines (RILs) and the near isogenic lines (NILs), respectively. These locations are representative sites of two crucial wheat production zones, Northern Winter Wheat Zone (I) and Yellow and Huai River Valleys Facultative Wheat Zone (II), which produce 68% of the current total wheat production in China (Wang *et al*., 2009, *Field Crops Res*. 111, 181-188.). The rest wheat production zones are Middle and Lower Yangtze Valleys Autumn-Sown Spring Wheat Zone (III), Southwestern Autumn-Sown Spring Wheat Zone (IV), Southern Autumn-Sown Spring Wheat Zone (V), Northeastern Spring Wheat Zone (VI), Northern Spring Wheat Zone (VII), Northwestern Spring Wheat Zone (VIII), Qinghai-Tibetan Plateau Spring-Winter Wheat Zone (IX) and Xinjiang Winter-Spring Wheat Zone (X).


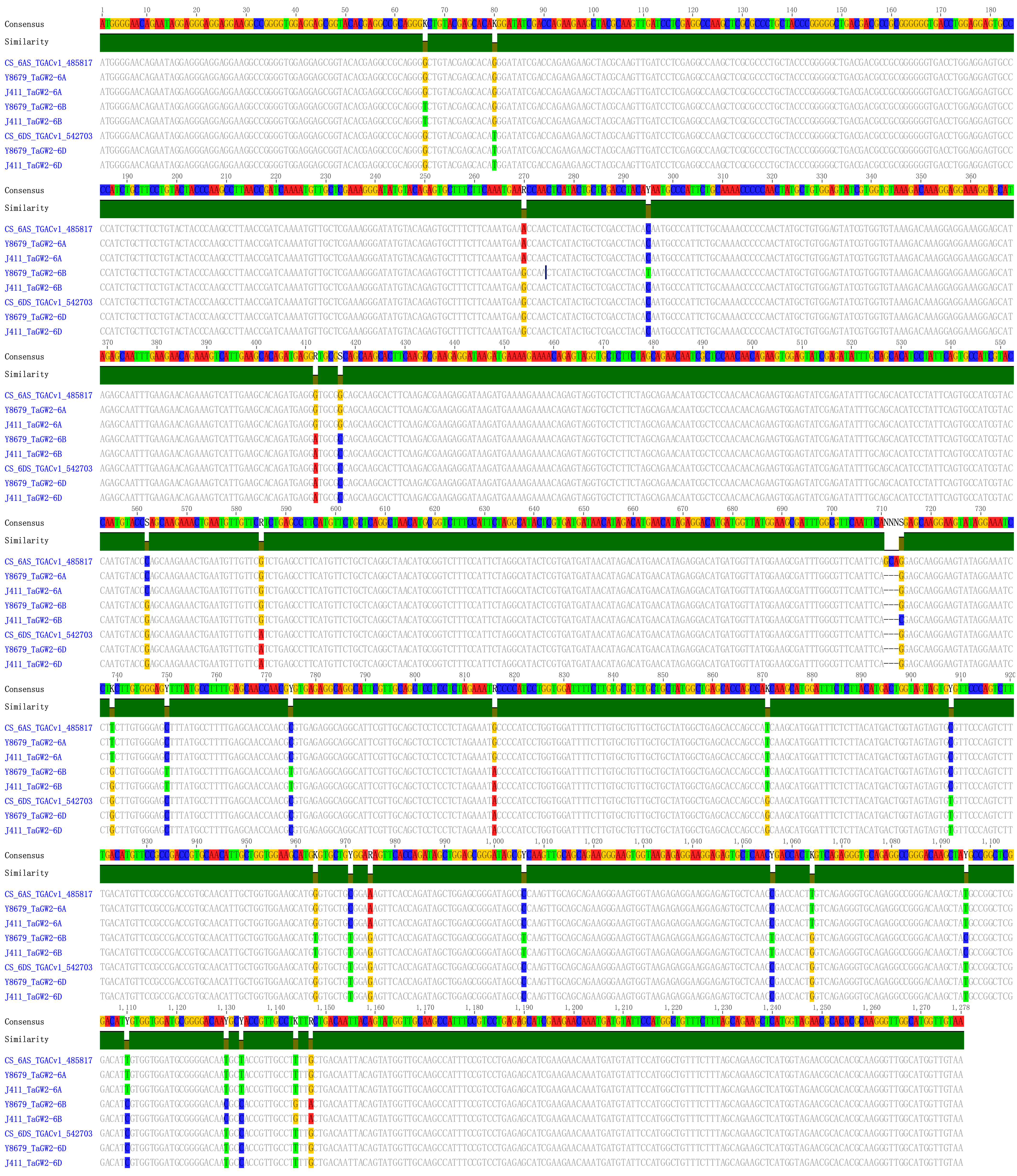


**Fig. S2** The coding sequences of three *TaGW2* homoeologs isolated from Y8679 and J411

**
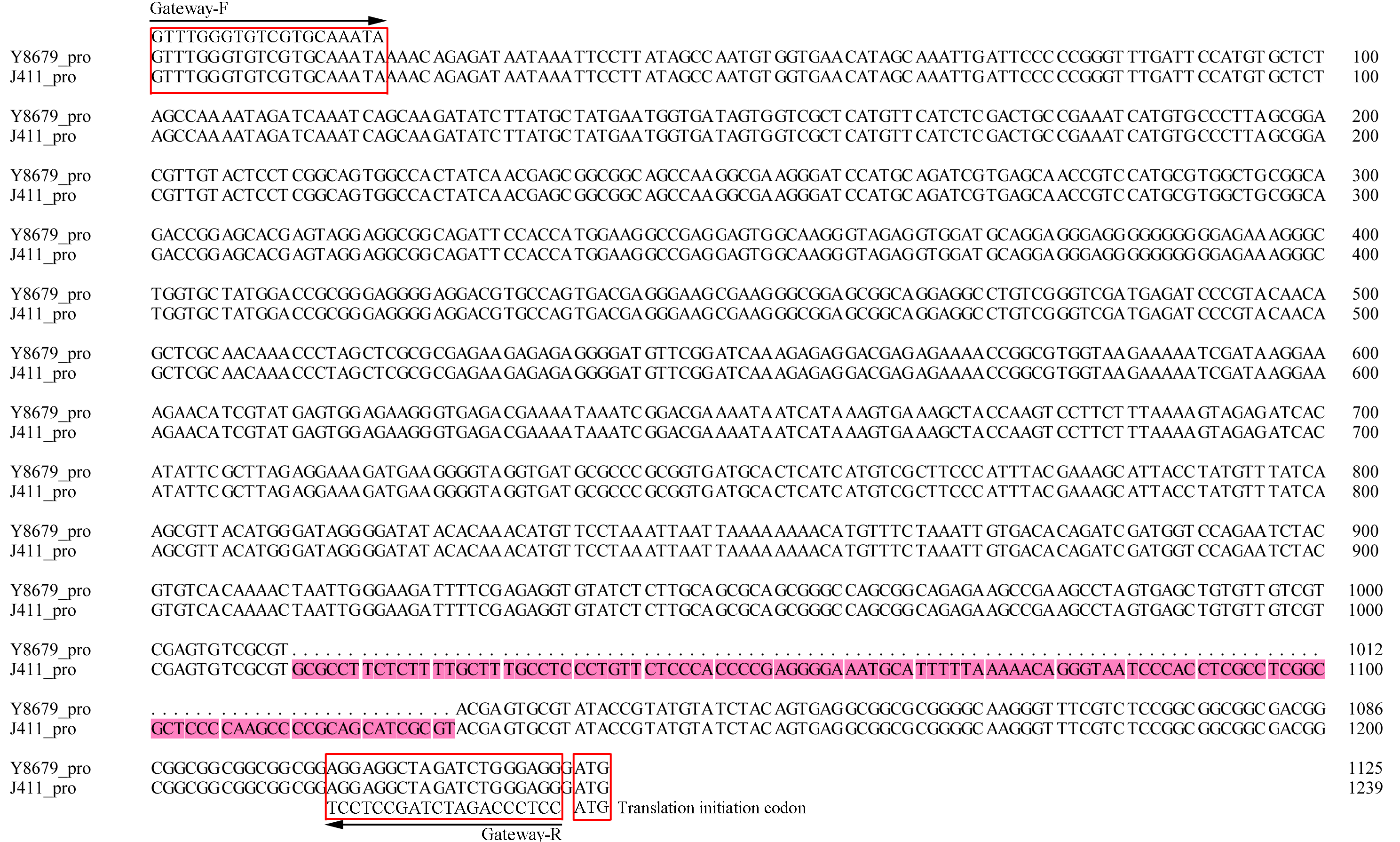
**

**Fig. S3** *TaGW2-A1* promoter sequences from Y8679 and J411 used for constructing promoter-GUS fusions. Corresponding primer positions and the translation initiation codon are indicated with red rectangles. The 114-bp InDel is colored in pink purple.


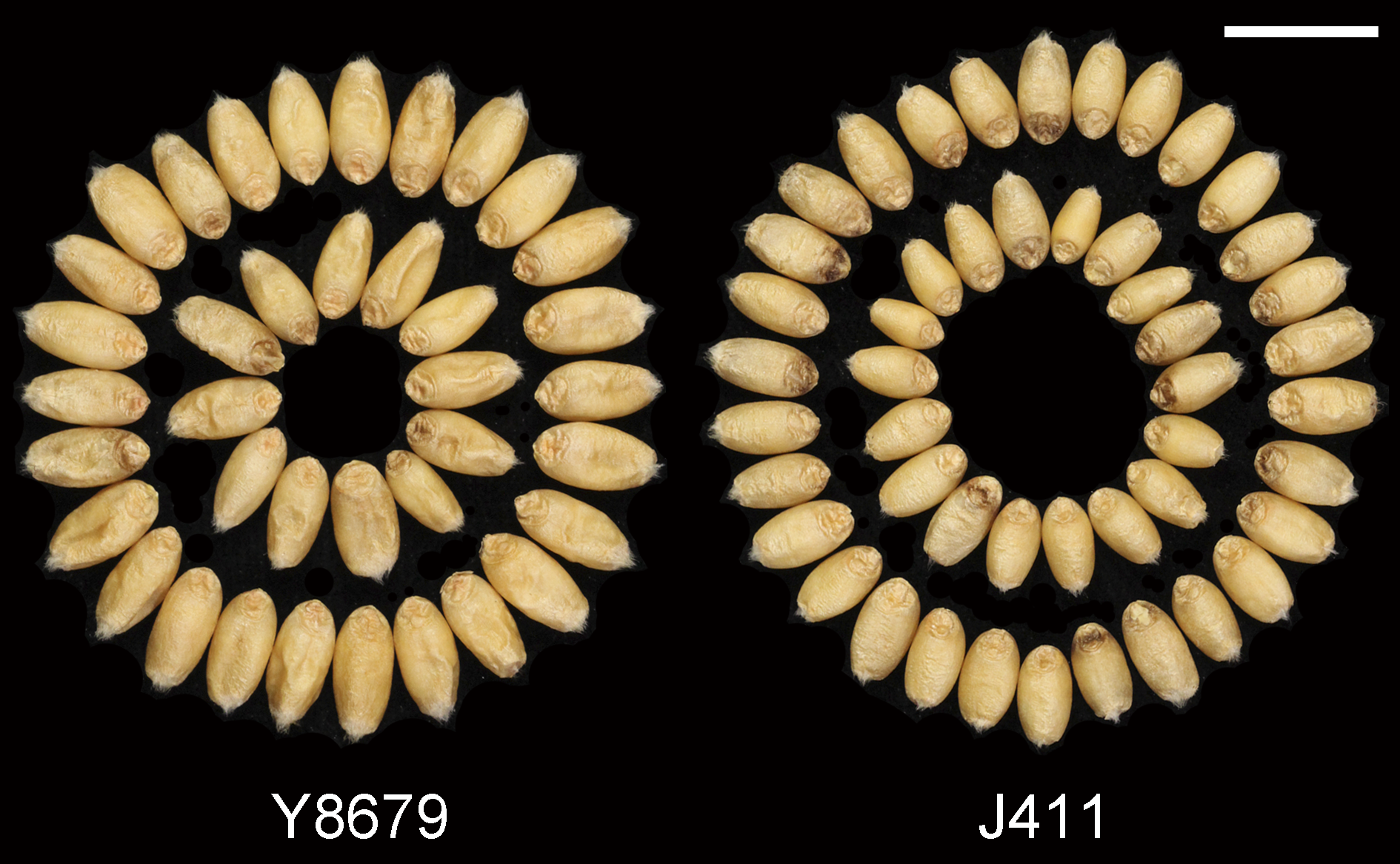


**Fig. S4** Grain size and number comparisons between Y8679 and J411. Seeds were collected from Shanxi at the 2013-14 growing season. Both groups of seeds were collected from a single spike. The bar represents 1 cm.


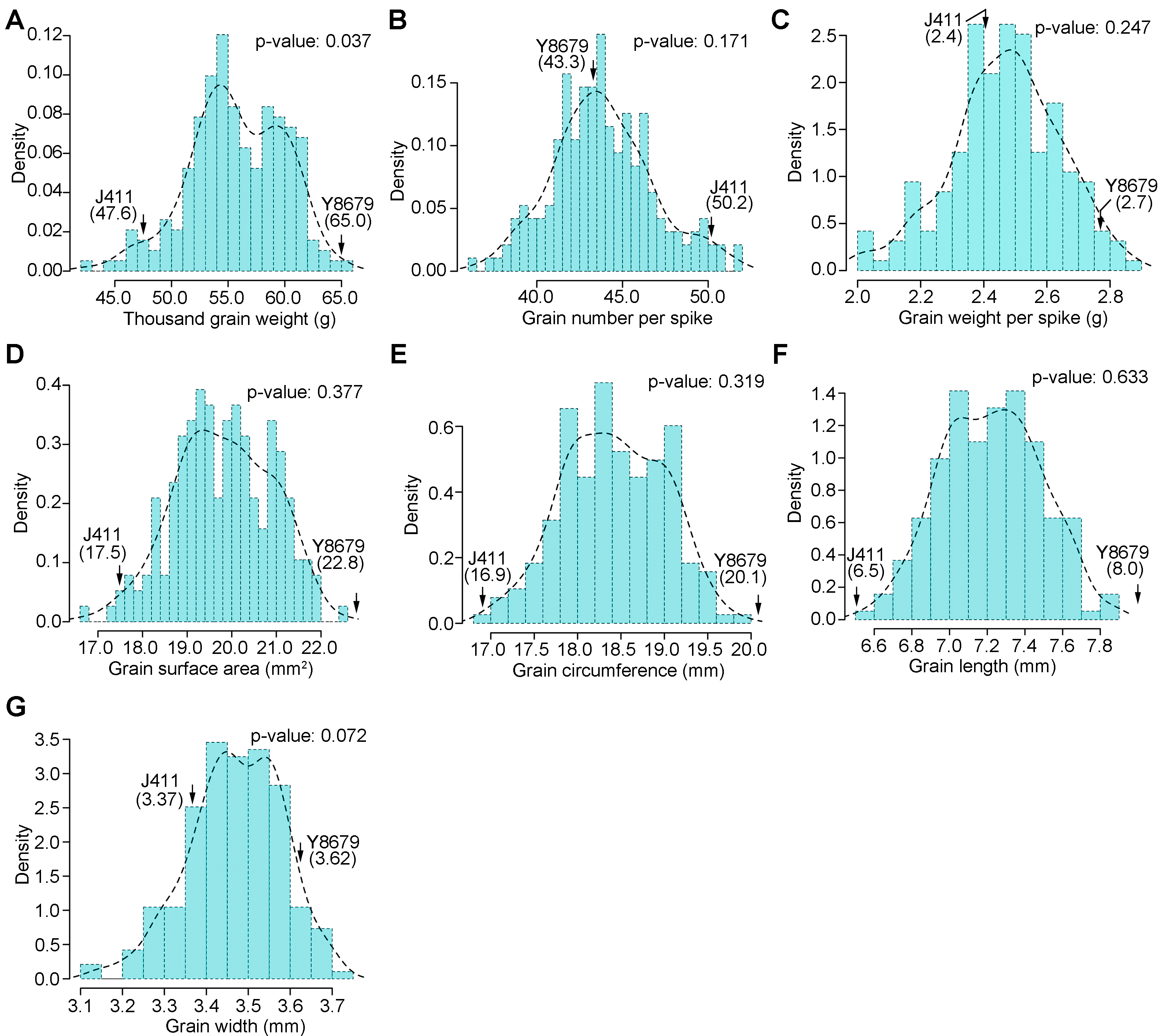


**Fig. S5** Histograms of the Y8679/J411 RIL population for thousand grain weight, grain number per spike, grain weight per spike and four grain morphometric parameters. p-value < 0.05 indicates a significant departure from the normal distribution (Shapiro-Wilk test).


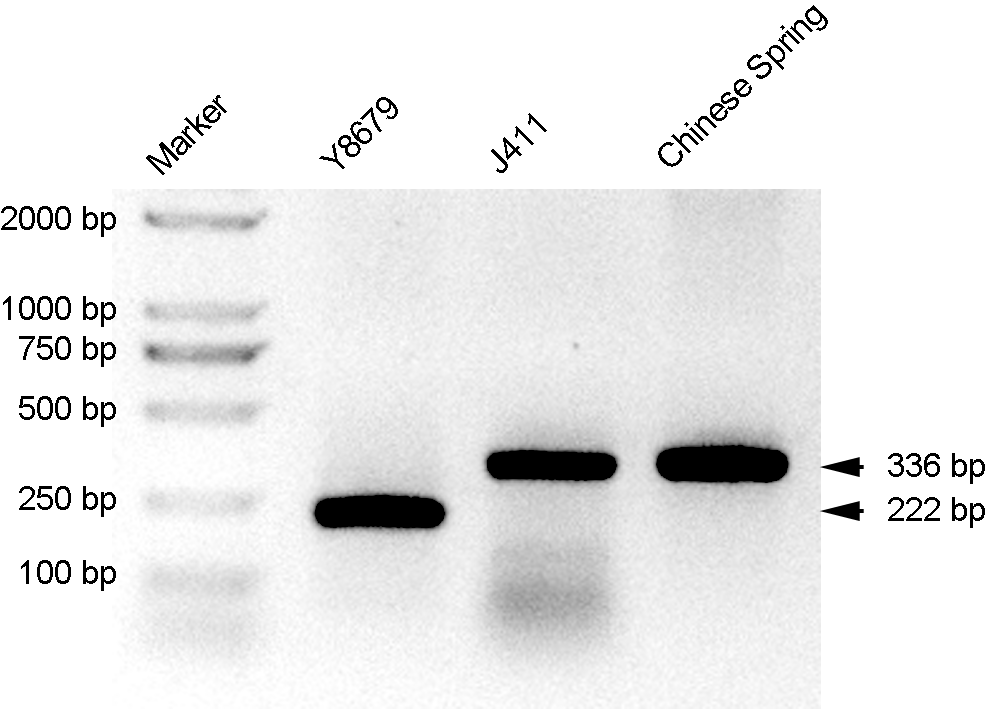


**Fig. S6** PCR products amplified from Y8679, J411 and Chinese Spring using primer pair TaGW2-A1_InDel

**
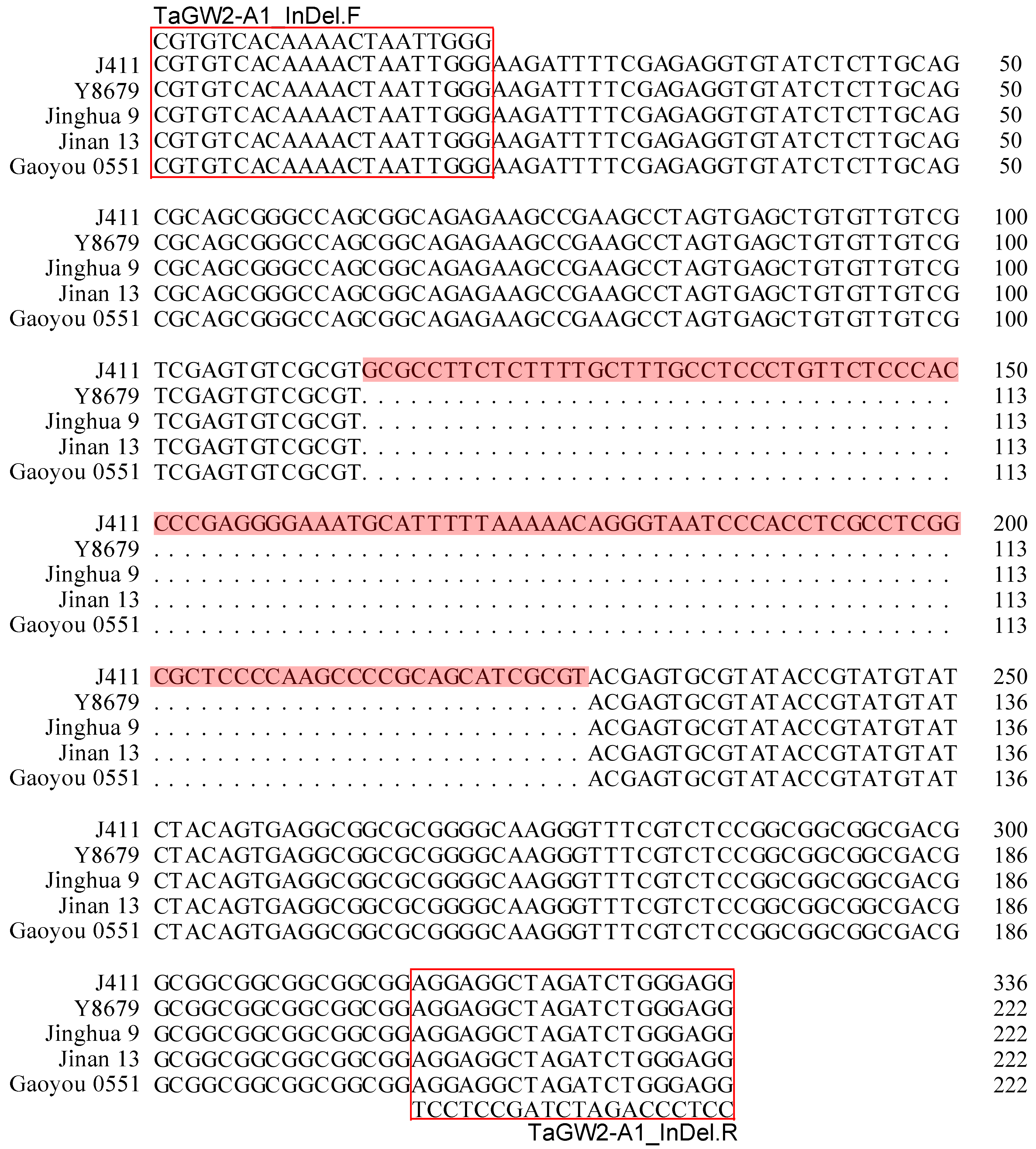
**

**Fig. S7** The sequences of PCR products amplified from Y8679, J411, Jinghua 9, Jinan 13, and Gaoyou 0551 with primer pair TaGW2-A1_InDel. Corresponding primer positions are indicated with red rectangles. The 114-bp InDel is colored in pink red.
